# Genome-Wide Investigation of Heat Shock Transcription Factor Family in Wheat (*Triticum aestivum* L.) and Possible Roles in Anther Development

**DOI:** 10.3390/ijms21020608

**Published:** 2020-01-17

**Authors:** Jiali Ye, Xuetong Yang, Gan Hu, Qi Liu, Wei Li, Lingli Zhang, Xiyue Song

**Affiliations:** College of Agronomy, Northwest A&F University, Yangling 712100, Shaanxi, China; yejiali@nwafu.edu.cn (J.Y.); yangxuetong@nwafu.edu.cn (X.Y.); huyunuo@163.com (G.H.); liuqi@nwafu.edu.cn (Q.L.); smallliwei@nwafu.edu.cn (W.L.); zhanglingli@126.com (L.Z.)

**Keywords:** evolution, genome-wide, heat shock transcription factor, thermo-sensitive male sterility, wheat

## Abstract

Heat shock transcription factors (HSFs) play crucial roles in resisting heat stress and regulating plant development. Recently, HSFs have been shown to play roles in anther development. Thus, investigating the HSF family members and identifying their protective roles in anthers are essential for the further development of male sterile wheat breeding. In the present study, 61 wheat HSF genes (*TaHsfs*) were identified in the whole wheat genome and they are unequally distributed on 21 chromosomes. According to gene structure and phylogenetic analyses, the 61 *TaHsf*s were classified into three categories and 12 subclasses. Genome-wide duplication was identified as the main source of the expansion of the wheat HSF gene family based on 14 pairs of homeologous triplets, whereas only a very small number of *TaHsfs* were derived by segmental duplication and tandem duplication. Heat shock protein 90 (HSP90), HSP70, and another class of chaperone protein called htpG were identified as proteins that interact with wheat HSFs. RNA-seq analysis indicated that *TaHsfs* have obvious period- and tissue-specific expression patterns, and the *TaHsfs* in classes A and B respond to heat shock, whereas the C class *TaHsfs* are involved in drought regulation. qRT-PCR identified three *TaHsfA2bs* with differential expression in sterile and fertile anthers, and they may be candidate genes involved in anther development. This comprehensive analysis provides novel insights into *TaHsfs*, and it will be useful for understanding the mechanism of plant fertility conversion.

## 1. Introduction

The heat shock response is common in plants where a series of stress responses are generated under heat stress [1]. As a consequence of heat stress, plant heat shock elements (HSEs) present in the promoter regions upstream of the heat shock protein (HSP) genes are recognized by the activated heat shock transcription factors (HSFs), and they induce the transcription of *Hsp* genes as molecular chaperones to help refold, assemble, distribute, and degrade related proteins, as well as repairing damaged proteins and maintaining cell survival [2,3,4]. In particular, regulation of the activity of HSFs is the key mechanism responsible for the transcription and expression of *Hsp*s.

A typical HSF has three components comprising the N-terminal DNA-binding domain (DBD), oligomeric domain (OD), and nuclear localization signal (NLS). Some HSFs also have a C-terminal activation domain (CTAD) and nuclear export signal (NES) [5]. DBDs are located at the N-terminus of HSFs and they are highly conserved regions with three helical structures (H1, H2, and H3) and four inverted parallel β-sheets (β1, β2, β3, and β4) [6]. The helix-turn-helix (H2-T-H3) structure at the hydrophobic center of the DBD allows the precise localization and recognition of HSE sequences (5′-AGAAnnTTCT-3′) [7]. The OD region is also located at the N-terminus of HSFs and it comprises two hydrophobic heptad repeat regions: A and B (HR-A/B). HR-A contains 5–6 sets of hydrophobic heptapeptide repeats, whereas HR-B has two overlapping heptapeptide repeats, which form a helical coiled-coil structure in space [8]. According to the characteristics of the HR-A/B region, plant HSFs can be classified into class A, class B, and class C. Class A and class C HSFs have 21 and 7 aa inserts between regions HR-A and HR-B, respectively, whereas class B HSFs are relatively simple with no amino acid insertions [9]. The synergy between the NLS and NES maintains the cell balance, thereby allowing plant HSFs to be freely distributed in the cytoplasm and nucleus [10]. Together with the NES, the CTAD acidic amino acid (AHA) motifs serve as a type-specific region in the C-terminus of class A HSFs in plants, which is characterized by aromatic and large hydrophobic amino acids [11]. By contrast, the B and C class HSFs have no activation functions due to the lack of an AHA domain [5].

The first HSF gene found in plants was cloned in tomato (*Solanum lycopersicum*) [12] and the HSF gene family have now been characterized in *Arabidopsis* (*Arabidopsis thaliana*), rice (*Oryza sativa*) [13], maize (*Zea mays*) [14], soybean (*Glycine max*) [15], chickpea (*Cicer arietinum*) [16], and sorghum (*Sorghum bicolor*) [17], as well as in vegetables including Chinese cabbage (*Brassica rapa*) [18] and pepper (*Capsicum annuum*) [19], and fruits such as apple (*Malus domestica*) [20] and pear (*Pyrus bretschneideri*) [21]. In addition to the *Hsf*s involved in the regulation of heat-resistance mechanism studied in *Arabidopsis* [22,23], many *Hsfs* have been found to participate in other responses in various plant species in recent years. The overexpression of *AtHsfA2* improves the tolerance of heat in *Arabidopsis* but it also enhances the salt tolerance [24], hypoxia resistance [25], and resistance to oxidative stress [26]. Under high temperature stress, the overexpression of *LeHsfA2* increases the fruit set rate in tomato and enhances fruit development [27]. Shim et al. found that the overexpression of *HsfA4a* in rice increased the tolerance to cadmium [28]. Almoguera et al. suggested that the overexpression of *HaHsfA9* alone or *HaHsfA9* together with *HaDreb2* can promote the synthesis and accumulation of HSPs in tobacco seeds and increase the seed life [29]. The heat-induced tomato *LeHsfB1* is a coactivator of class A *Hsfs* (e.g., *HsfA1a*) and it has an auxiliary activation function by enhancing the expression of target genes [30]. However, studies have rarely shown that class C *Hsfs* can be induced by various stresses [18].

Higher temperature directly leads to a decreased number of spikes per ear and spikelets per spike, and a lower grain weight in wheat [31]. There is no doubt that improving the heat tolerance of wheat and ensuring adequate food production are vital for humans. Members of the HSF family have been identified in wheat based on the old reference genome [32], but a lack of sufficient information has hindered in-depth studies of the stress resistance mechanisms related to *TaHsfs*. A limited number of *TaHsfs* have been functionally analyzed, and studies have demonstrated that *TaHsfA4a*, *TaHsfA6e*, *TaHsfB2d*, and *TaHsfC2a* function as wheat HSF genes to enhance the tolerance of heat, confer strong cadmium tolerance, and participate in protecting the grains [28,33,34,35]. Therefore, a more comprehensive and systematic analysis is required of *TaHsf*s based on the newly published wheat reference genome in order to provide a reliable basis for elucidating their functions. 

The importance of heterosis utilization in increasing plant yield is self-evident. The successful use of two-line hybrid rice has effectively proved the great advantages of photo/ thermo-sensitive male sterility in practical production. The thermo-sensitive male-sterile wheat KT3315A obtained by our research team can be used for selfing breeding under high temperature conditions and for cross breeding under low temperature conditions, which greatly facilitates wheat breeding procedures and hybrid production. The fertility conversion of thermo-sensitive male sterile wheat is controlled by temperature; therefore, understanding its temperature response mechanism is the first task to understand the fertility conversion mechanism. Significant differences in microspore development and anther appearance in sterile and fertile KTM3315A have been verified [36]. In addition to genes that synthesize nutrients necessary for anther and microspore development, we found that *Hsfs* and *Hsps* were differentially expressed under different fertility conditions (different environmental temperatures during the wheat growing season), and thus we speculated that they may be related to anther development [37]. The *Hsfs* on the sex chromosomes of humans and animals have been shown to affect spermatogenesis [38,39]. Coincidentally, in recent years, the HSFs involved in male reproductive cell protection have also attracted attention in plants. In the rice spike, eight HSF genes are up-regulated in anther development [40]. In addition, the up-regulated expression levels of *HsfA2* and *HsfA3* in tomato pollen grains demonstrate their involvement in anther development [27,41]. However, the HSF gene family members involved in anther protection have not yet been reported in wheat. Therefore, it is necessary to comprehensively investigate the HSF gene family in wheat and explore their possible protective roles in anther development.

In this study, we comprehensively analyzed the wheat HSF family based on the latest release of the complete wheat reference genome. We identified the members of the HSF gene family in wheat as well as investigated their evolutionary relationships, gene structures, and expression levels. Our findings may facilitate subsequent studies of the stress resistance mechanism in wheat, as well as provide a reference for understanding molecular temperature sensing and regulation mechanisms in plants with temperature-sensitive biological characteristics.

## 2. Results

### 2.1. Identification and Characteristics of TaHsfs

The newly constructed wheat-specific hidden Markov model (HMM) file was used to search the whole wheat protein sequences and 94 candidate Hsfs were obtained, which were consistent with the wheat candidate Hsfs obtained by aligning with *Arabidopsis* and rice Hsfs. After excluding candidate genes without a DBD domain and coiled-coil structure, 61 non-redundant genes were finally identified as wheat HSF family members (Table S1, Table S2). These 61 TaHsfs were found to be unequally distributed on 21 chromosomes (Figure S1), where the 5A chromosome contained the most *TaHsfs* (6) and one to five *TaHsfs* were unevenly distributed on the other chromosomes. These HSF genes identified in wheat encode proteins ranging from 227 to 569 amino acids (aa) with an average of 360 aa. The molecular weights of the TaHsfs vary from 24.69 (TaHsfC1b-3) to 59.76 (TaHsfB2c-1), and the predicted isoelectric points range from 4.85 (TaHsfA2b-2) to 9.52 (TaHsfB1a-3) (Table S3).

### 2.2. Evolution and Phylogenetic Analysis of TaHsfs

**Figure 1:**
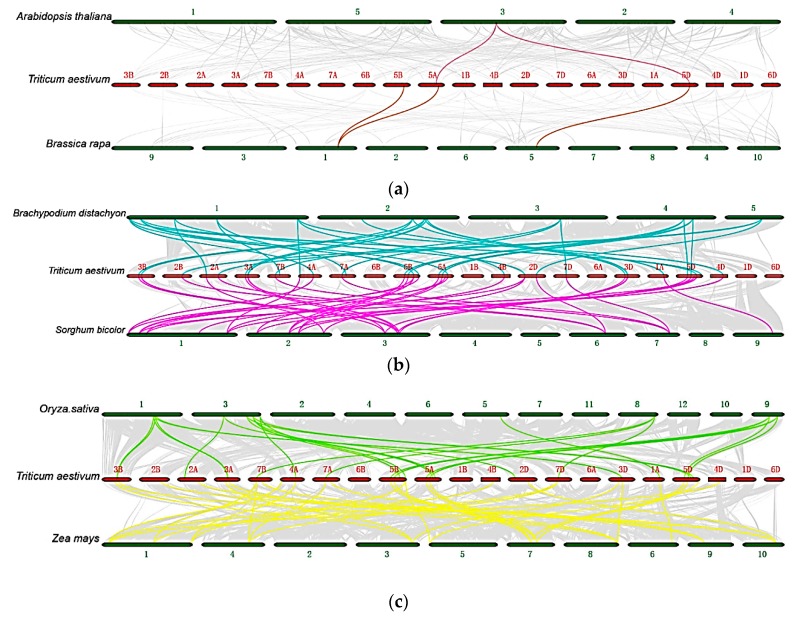
Synteny relationships of *Hsfs* in wheat and six representative species. (**a**) Synteny relationships of *Hsfs* between wheat with *Arabidopsis* (*Arabidopsis thaliana*) and Chinese cabbage (*Brassica rapa*). (**b**) Synteny relationships of *Hsfs* between wheat with rice (*Oryza sativa*) and maize (*Zea mays*). (**c**) Synteny relationships of *Hsfs* between wheat with sorghum (*Sorghum bicolor*) and *Brachypodium* (*Brachypodium distachyon*). Gray lines in the background indicate the synteny blocks for wheat and other plant genomes, and the lines in other colors highlight the synteny of *Hsf* pairs.

In two different species, the conservation of the position and order of homologous genes at a locus is called synteny of the genome [42]. If the species are closer, the genome coverage of the synteny fragment is the larger and it will contain more genes. To determine the evolutionary trends and genetic relationships in the wheat HSF gene family, we performed synteny analysis between wheat and *Arabidopsis*, Chinese cabbage, rice, maize, sorghum, and *Brachypodium* (*Brachypodium distachyon*). The results showed that *TaHsfs* are distantly related to the *Hsfs* in *Arabidopsis* and Chinese cabbage, with only two and three HSF genes within the synteny segment, respectively (Figure 1A). By contrast, the number of HSF genes in synteny were higher between wheat and rice (25), sorghum (39), maize (30), and *Brachypodium* (34) (Figure 1B,C), which all belong to the Gramineae. *Arabidopsis* and Chinese cabbage are both cruciferous, so 21 HSF genes were within the synteny segment (Figure S2). After the differentiation of gramineous species, synteny at the entire chromosome level was found [43]. In this study, the HSF gene family in wheat and other gramineous plants also exhibited closer kinship. Clearly, our results indicate that the HSF gene family remains stable and the evolution of *Hsfs* is consistent with that of the whole genome. 

**Figure 2:**
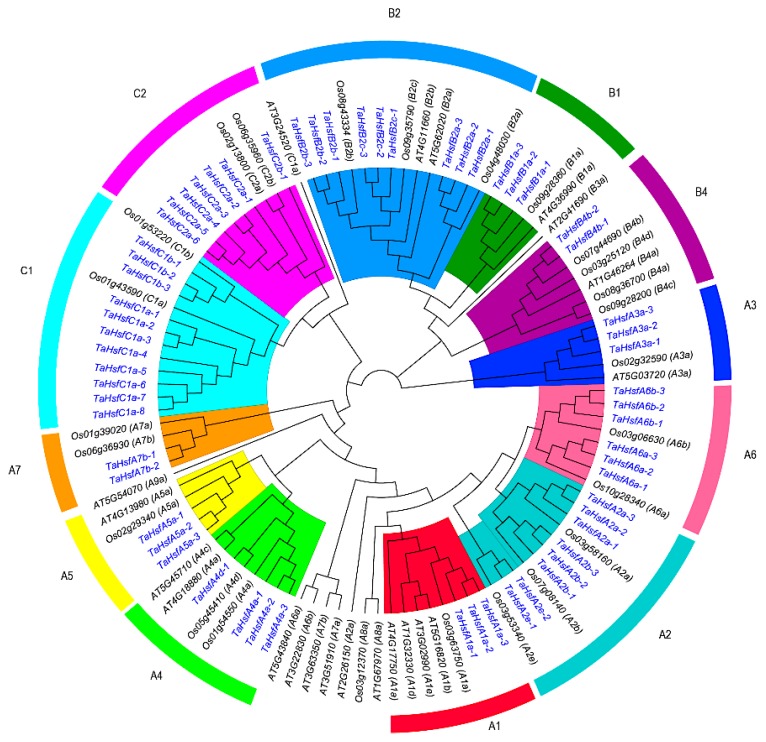
Phylogenetic tree obtained for the heat shock transcription factor (HSF) family members in wheat, rice, and Arabidopsis. Different colors represent different sub-classes in the HSF gene family.

In order to clarify the classification and evolutionary status of the members of the wheat HSF gene family, 108 full-length amino acid sequences HSF in the related species rice (25), and model plants *Arabidopsis thaliana* (22) and wheat (61) were used to construct a phylogenetic tree (Figure 2). Based on the clustering results, 61 *TaHsfs* were classified into three major categories: A, B, and C. The *TaHsfs* in class A belonged to seven subclasses comprising A1–A7; the class B *TaHsfs* were divided into three subclasses comprising B1, B2, and B4; and the class C *TaHsfs* were divided into two subclasses comprising C1 and C2. Compared with the rice and *Arabidopsis* HSF gene families, the wheat HSF gene family lacks members belonging to A7, A8, A9, and B3. Few orthologous genes were detected in the HSF family members between *Arabidopsis* and wheat. However, in every subclass, at least one rice HSF was found to be highly homologous with the wheat HSFs, and almost every rice HSF was shown to be orthologous with three wheat HSFs. Therefore, the number of wheat HSFs is close to or equal to three times that in rice HSF in terms of the total number and the number in each subclass. 

### 2.3. Duplication Events in Wheat HSF Family

To understand the source of the larger number of *TaHsfs*, we analyzed the causes of the expansion of the wheat HSF family. We performed homeologous group analysis for the wheat HSF family based on the homologous alignment within the wheat HSF family, the distribution of each gene on the chromosomes, and clustering in the phylogenetic tree. As expected, 68.8% of the wheat HSFs exhibited homology of 1:1:1, where three *TaHSFs* localized on the three A, B, and D sub-genomes shared high homology, which we refer to as triplets (Table 1, Table S4). The proportion of homeologous triplets in the wheat HSF family was close to twice the proportion of homeologous triplets in the entire wheat genome (35.8%) [44]. This high proportion indicates that wheat polyploidization was the main cause of the expansion of the wheat HSF family, and the triplets remained stable during the long evolutionary process. However, gene losses (1:0:1/1:1:0) occurred in a very low number of triplets, where the proportion of losses was close to that in the whole wheat genome, and such losses are usually induced by function redundancy. Further investigation is need to understand more complex phenomena (orphans/singletons and others), and the expansions in C1 and C2 classes indicated that some other gene duplication events occurred in the wheat HSF gene family.

**Figure 3:**
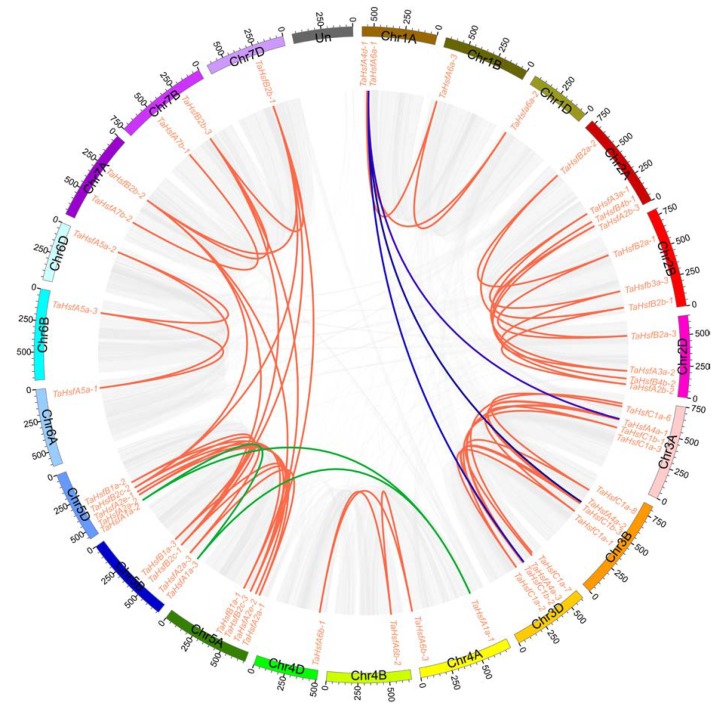
Genomic locations of *Hsfs* and segmental duplicated gene pairs in the wheat genome. Gray lines in the background indicate the synteny blocks within the whole wheat genome, and red lines denote the segmental duplication HSF gene pairs. The blue lines show genes that have undergone segmental duplication with *TaA4d-1*, while the green lines show the genes that have undergone segmental duplication with *TaA1a-1*.

We performed synteny analysis to further investigate the sources of other *TaHsfs*, i.e., the causes of wheat HSF gene family expansion in addition to genome-wide duplication. Figure 3 shows that all *TaHsfs* derived from genome-wide duplication were involved in segmental duplication events (Table S5), which probably led to the high homology of the A, B, and D sub-genomes in wheat. The paralogues *TaHsfA1a-1*, *-2*, and, *-3* located on the 4A, 5B, and 5D chromosomes, respectively, were derived from segmental duplication events. Interestingly, the orphan gene *TaHsfA4d-1* (Chr1A) identified above was a segmental duplicate of *TaHsfA4a-1*, *-2*, and *-3* (Chr3A, 3B, and 3D). In addition, two pairs of tandem duplications comprising *TaHsfC1a-6*/*TaHsfC1a-4* and *TaHsfC1a-7*/*TaHsfC1a-5* occurred in the wheat HSF gene family on the 3A and 3D chromosomes, respectively (Figure S1), and their KA/KS values were 0.367731 and 0.388683. The analysis of these two duplication events complemented the sources of the four genes described above (*A1a-a*, *A4d-1*, *C1a-4*, *C1a-5*), but the seven *TaHsfs* in the C2 subclass were unexpectedly not present in either case. The unusual features of the C2 sub-class members indicated that further analysis of their structure is required.

### 2.4. Gene Structure and Motif Composition in TaHsfs

**Figure 4:**
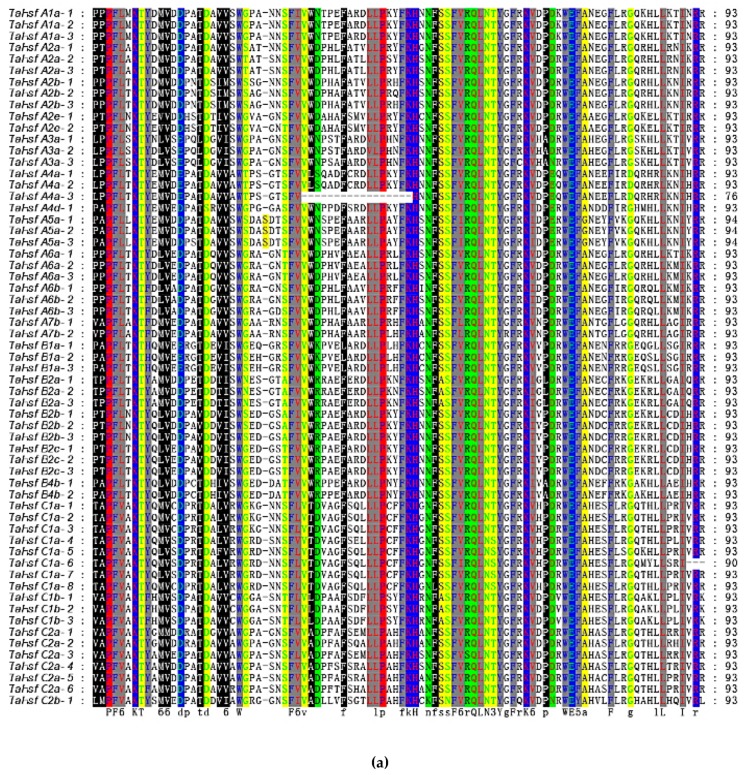

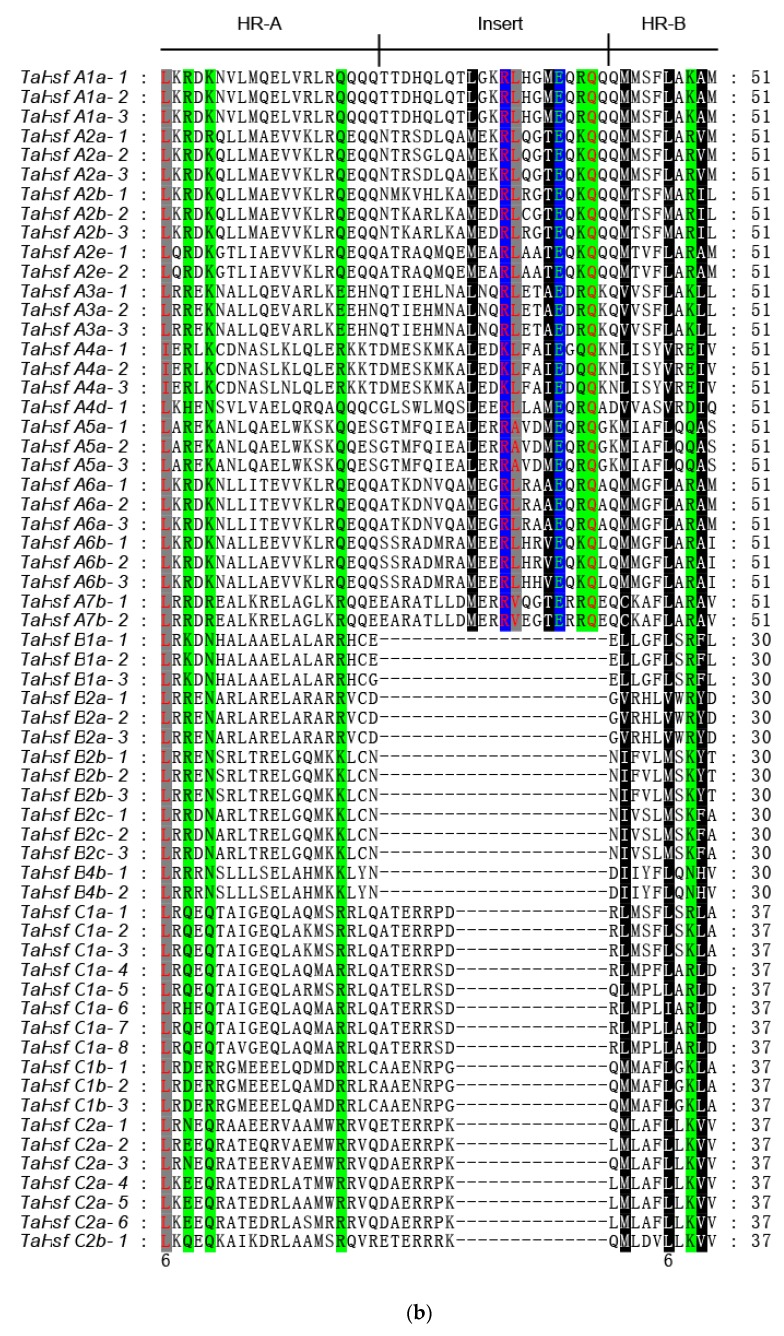
Analysis of the conserved DNA-binding domain (DBD) domains and oligomeric domain (OD) regions in the 61 *TaHsfs*. (**a**) Multiple sequence alignment of the DBD domains in 61 *TaHsfs*. (**b**) Multiple sequence alignment of the heptad repeat A/B (HR-A/B) regions in 61 *TaHsfs*. The scheme at the top shows the locations and boundaries of the HR-A core, insert, and HR-B regions within the HR-A/B regions.

**Figure 5:**
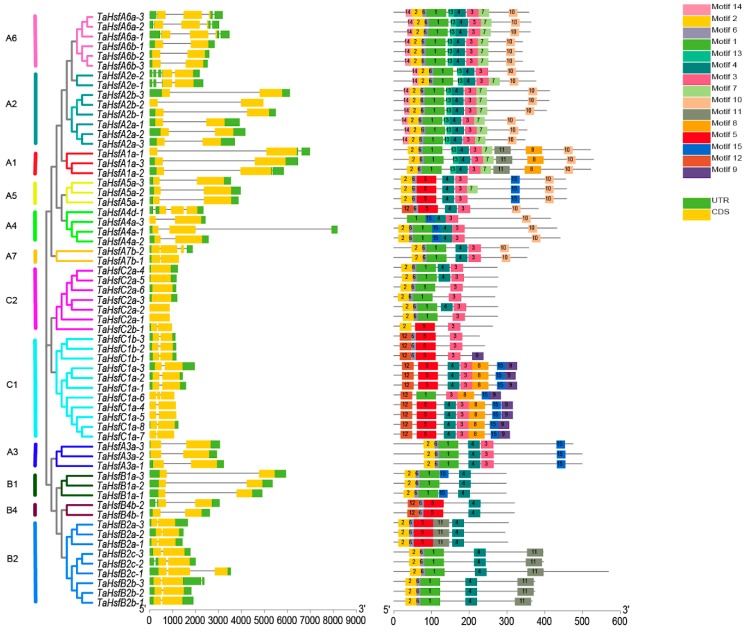
Phylogenetic tree, gene structure, and architecture of conserved protein motifs in *TaHsfs.* Details of the clusters in the phylogenetic tree are shown in different colors. The left panel represents the intron–exon structure of the *TaHsfs*, and the right panel shows the motif compositions of the *TaHsfs*. Different motifs are indicated by different colors and they are numbered from 1–15.

Multiple sequence alignments were performed to further analyze the degree of conservation in the DBD and OD domains in the *TaHsfs*. The results showed that the DBD amino acid lengths in the *TaHsfs* were 76–94 aa (Figure 4A). In particular, *TaHsfA4a-3* (76 aa) had a 17-aa deletion compared with most of the *TaHsfs (93aa)*. According to the OD structure, the classification of the gene family was consistent with the results obtained by phylogenetic analysis, with 21 and 7 aa insertions between HR-A and HR-B in the class A and class C *TaHsfs*, respectively, and no insertions between HR-A and HR-B in the class B *TaHsfs* (Figure 4B). The positional information for the conserved DBD, OD, NLS, NES, and AHA domains showed that the same conserved domains in the same sub-class are almost identical, e.g., each conserved domain has exactly the same position and sequence among the three members of the A5 subclass (DBD: 17–110, OD: 138–188, NLS: 210-DALHKKRRLSGLDY, and AHA: 414-DNFWEQFLTE) (Table S6). Figure 5 shows that the number of introns is small in the *TaHsfs*, ranging from 0 to 4, but the lengths of the introns vary greatly. Motif prediction identified 15 conserved motifs with high confidence ranging between sizes of 11–50 aa in the *TaHsf* family, where motif 1 was the longest (50 aa) and motif 6 was the shortest (Table S7). Motifs 1, 2, 5, 6, 12, and 14 correspond to the DBD domain, and their highly conserved structures make them highly compatible with HSEs to ensure their accurate localization and the precise regulation of downstream gene expression [7]. Motifs 3 and 4 correspond to the OD domain, and motif 10 corresponds to the AHA motif. Structural analysis demonstrates that the *TaHsfs* in the C2 subclass belong to the wheat HSF gene family because they have the unique sequence characteristics of the C class but also the same conserved motifs as other members.

### 2.5. Interaction Network Analysis

**Figure 6:**
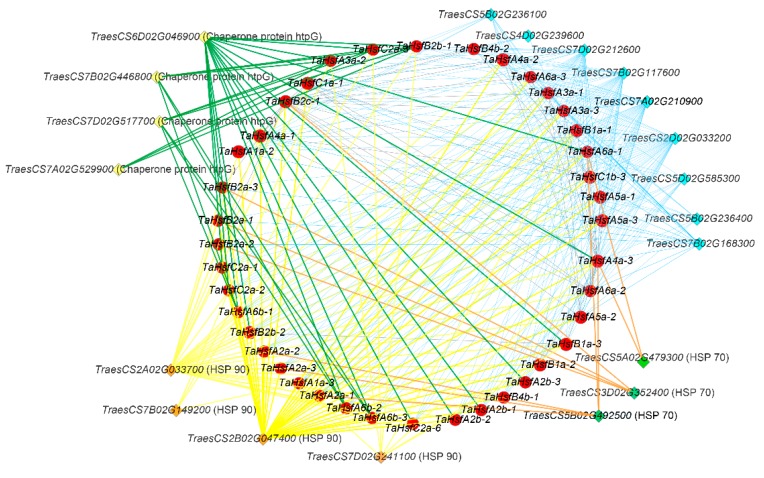
Interaction network between *TaHsfs* and related genes. The red circles represent *TaHsfs* and the diamonds in other colors represent the genes that interact with them. Lines connecting two genes show that an interaction exists between them.

Interaction network analysis can help to understand the biological functions and molecular mechanisms of proteins. Therefore, we predicted the proteins that interact with wheat HSFs using STRING (https://version-10-5.string-db.org/cgi/input.pl?sessionId=bHwqHDUnAsoF&input_page_show_search=on). The results showed that there were many interactions between TaHsfs, and proteins encoded by many other genes not belonging to this family also interacted with TaHsfs. HSP70s, HSP90s, and another class of chaperone protein called high-temperature protein G (htpG) were identified as the three major types of proteins that interact with wheat HSFs (Figure 6). In particular, HSP70 is an interesting and highly conserved protein, and the insertion of an HSP70 antisense gene fragment in sorghum and rice has been confirmed to lead to pollen abortion and male sterility [45,46]. 

### 2.6. Expression Profiles of TaHsfs

**Figure 7:**
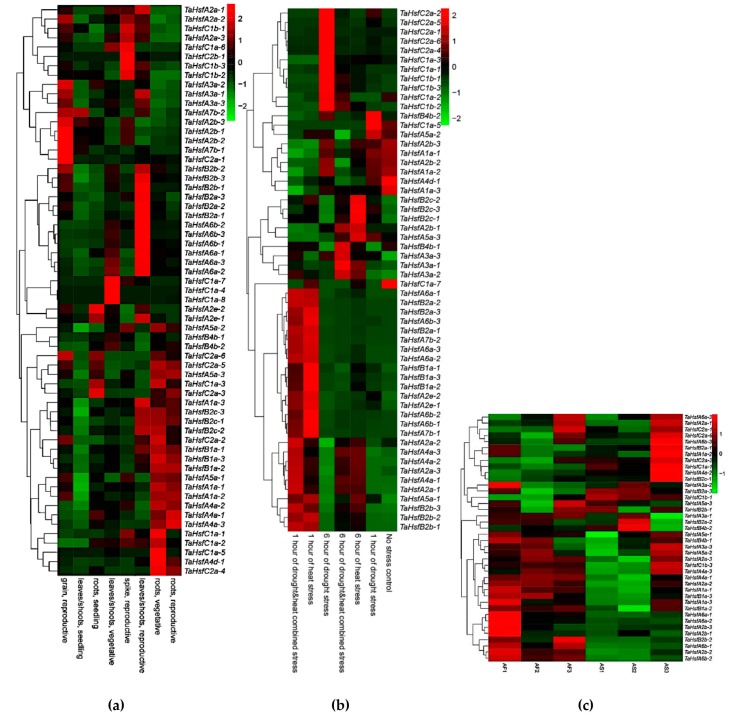
Expression profiles for *TaHsfs* in different conditions. (**a**) Heat map of *TaHsfs* in nine different tissues and growth stages under normal conditions. (**b**) Heat map of *TaHsfs* under five different abiotic stress conditions and in a normal control. (**c**) Heat map of *TaHsfs* in the thermosensitive male sterile wheat KTM3315A at two different temperatures. Heatmap obtained using the count values from RNA-seq analysis.

In order to fully understand the expression patterns of the *TaHsfs* in different tissues and during different growth periods, we analyzed RNA-seq data from the roots, stems, and leaves in the seedling, vegetative growth, and reproductive growth stages, as well as spikes from the reproductive growth stages, and the mature grain in Chinese Spring wheat (Wheat Expression Browser, http://www.wheat-expression.com/, developmental time-course of Chinese Spring). Except for the similar expression patterns of *TaHsfs* in the roots during reproductive growth and vegetative growth, the expression levels of *TaHsfs* differed in the other periods and tissues (Figure 7A, Table S8). Two specific *TaHsfs* (*C1a-4, C1a-8*) were expressed in the stems and leaves in the reproductive stage, and they were not expressed in the other tissues and periods. In particular, seven *TaHsFs* (*A2a-2, C1b-1, A2a-3, C1a-6, C2b-1, C1b-3, C1b-2*) were up-regulated in the spikes.

In addition, we analyzed the expression levels of *TaHsfs* under different physiological conditions using RNA-seq data from the expVIP database (Wheat Expression Browser, http://www.wheat-expression.com/genes/heatmap, drought and heat stress time-course in seedlings). The heatmap showed that compared with almost all *TaHsfs* of the no stress control, almost all *TaHsfs* did not exhibit differences in their expression levels in response to drought stress for 1 h, but 10 members of the C1a, C2a, and C1b sub-classes of *TaHsfs* were significantly up-regulated under drought stress for 6 h. Under heat stress for 1 h and the combination of heat stress and drought stress for 1 h, the expression levels of the *TaHsfs* were similar, where the main up-regulated *TaHsfs* belonged to classes A and B. *TaHsfs* from B2c and A3a were up-regulated under heat stress for 6 h and the combination of heat stress and drought stress for 6 h, respectively (Figure 7B, Table S9). Our analysis showed that class A and B *TaHsfs* were significantly up-regulated under heat stress, whereas class C *TaHsfs* were significantly up-regulated under drought stress. Subclass B1, B2, and B4 *Hsfs* were induced by the heat shock reaction, and they may act as synergistic factors that allow A class *Hsfs* to resist heat more effectively [30,47,48]. 

To determine the responses of *TaHsfs* in thermo-sensitive male sterile wheat to different fertility conditions, we analyzed the RNA-seq data for 40 *TaHsfs* under two fertility conditions in three different periods (uninucleate, binucleate, and trinucleate stages). We designated the sterile and fertile anthers in these three periods as AS1–3 and AF1–3, respectively. The results showed that compared with the sterile conditions, eight *TaHsfs* from A6a (-1, -2), A2b (-1, -2, -3), A6b (-1, -2), and B2b (-2) were always up-regulated in AF. However, no *TaHsfs* in subclass C were up-regulated under fertile conditions during these three stages (Figure 7C, Table S10). 

### 2.7. Validation of TaHsfs Involved in Anther Protection Mechanisms

**Figure 8:**
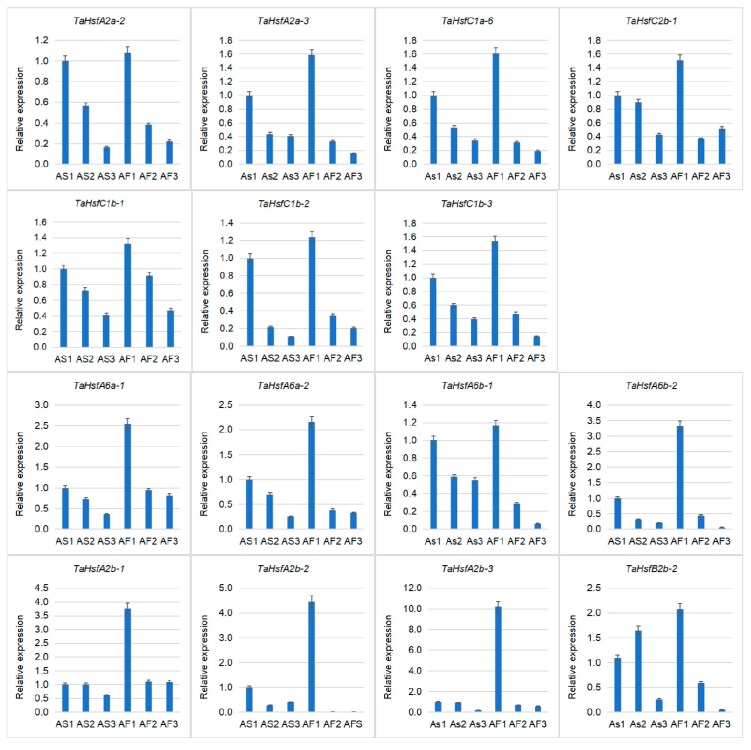
qRT-PCR results for *TaHsfs*. up-regulated *TaHsfs*in the spikes of Chinese spring wheat according to RNA-seq: *TaHsfA2a-2*, *TaHsfA2a-3*, *TaHsfC1a-6*, *TaHsfC1b-1*, *TaHsfC1b-2*, *TaHsfC1b-3*, and *TaHsfC2b-1*. Up-regulated *TaHsfs* in the fertile anthers of KTM3315A according to RNA-seq: *TaHsfA6a-1*, *TaHsfA6a-2*, *TaHsfA2b-1*, *TaHsfA2b-2*, *TaHsfA2b-3*, *TaHsfA6b-2*, *TaHsfA6b-1*, and *TaHsfB2b-2*. The x-axis represents six materials comprising sterile anthers and fertile anthers from the uninucleate, binucleate, and trinucleate stages. The y-axis represents the relative expression levels of *TaHsfs*. All data are the means ± SE of three independent experiments.

Based on the RNA-seq data, we identified seven up-regulated genes in the spikes of Chinese Spring and eight up-regulated genes in the fertile anthers of KTM3315A. In order to identify differentially expressed *TaHsfs* in fertile and sterile anthers, we performed quantitative real-time PCR (qRT-PCR) for the 15 candidate *TaHSfs* in three stages of anther development. The results showed that the expression levels of all 15 *TaHsfs* were highest in the uninucleate stage and they gradually decreased in the following two stages (binucleate stage and trinucleate stage) (Figure 8). There was no quantitative difference in the expression levels of the seven up-regulated *TaHsfs* in the Chinese Spring spikes during the three stages for AS and AF. However, the qRT-PCR results for the other eight *TaHsfs* were consistent with the RNA-seq results, where they were up-regulated in the uninucleate stage for AF compared with AS, and three *TaHsfA2bs* (-1, -2, -3) were significantly up-regulated. The regulation of *HsfA2* genes is known to be involved with redox homeostasis and metabolic, which are critical pathways for normal anther development [49]. In addition, the expression levels of *HsfA2s* are high in the early stages of anther and pollen development, and their important roles in the regulation of pollen protection genes have been confirmed in tomatoes [27,40,50]. Therefore, the three *TaHsfsA2bs* with high level expression levels in the fertile anthers may be candidate genes involved in anther protection during fertility conversion in the thermo-sensitive male sterile wheat line KTM3315A.

## 3. Discussion

Wheat is an important food crop throughout the world, and thus increasing its stress resistance, yield, and quality are of great economic importance. The release of the wheat genome sequence has provided important information to facilitate the identification of excellent agronomic traits and stress resistance genes in wheat at the genome level, thereby contributing to develop high quality and stress-resistant wheat varieties. 

In plants, HSFs are involved in various molecular processes and they have essential roles in the responses to abiotic stresses. Previous studies identified 21, 25, 25, 25, 25, and 22 *Hsf* genes in *Arabidopsis*, rice, maize, sorghum, tomato, and chickpea, respectively [13,14,15,16,51]. The number of HSF family members appears to be unrelated to the genome size, e.g., 35 *Hsfs* were found in Chinese cabbage (485 Mb) and 137 *Hsfs* in pear (527 Mb) [21,52]. Previous studies identified 56 and 82 members of the wheat HSF family [32,53], but we identified 61 *TaHsfs* in this study. To understand these differences in the numbers of wheat HSF members identified, we blasted the protein sequences identified by Xue et al. [32] and Duan et al. [53] against the latest wheat reference genome (IWGSC V1.1) [43]. Using the latest version gene IDs as standards, 77 genes were identified in all three studies, where four unique genes were identified in the present study, whereas six unique genes were identified by Duan et al. [53] and one by Xue et al. [32] (Figure S3, Table S11). The 16 protein sequences not identified as HSFs in the present study were analyzed. Although the DBD domains were detected, no high threshold coiled-coil structure was found by using MARCOIL software (https://bcf.isb-sib.ch/webmarcoil/webmarcoilC1.html) [54]. These findings matched with the identification criteria employed in the present study, so they were excluded. In total, 41 common HSFs were identified in all three studies, but the classifications and names of some of these proteins differed in the studies by Xue et al. [32] and Duan et al. [53]. For example, *TraesCS1A02G375600*, *TraesCS1D02G382900*, and *TraesCS1B02G396000* were renamed as *TaHsfA6a-1*, *TaHsfA6a-2*, and *TaHsfA6a-3* in the present study because they belong to a homeologous group and are orthologous to rice *Os10g28340* (A6a). However, Duan et al. [53] classified these genes in classes A4 and C1, and *TraesCS1B02G396000* was not even identified in the study of Xue et al. [32]. The main reasons for the differences in the three studies are as follows. First, the wheat genome sequence was not completed when these previous studies were conducted, and thus the assembly of incomplete and incorrect genes resulted in the failure to find all of the HSF gene family members. Second, the different threshold settings and strategies employed as identification criteria resulted in differences in the number of wheat HSF family members identified.

In the present study, in addition to employing two approaches described in the method section for identifying *TaHsfs*, we used the wheat HSF sequence in the plantTFDB database (http://planttfdb.cbi.pku.edu.cn/family.php?sp=Tae&fam=HSF) and NCBI (https://www.ncbi.nlm.nih.gov/) as query sequences to search for all of the wheat protein sequences. However, after verification based on Pfam (http://pfam.xfam.org/) [55], SMART (http://smart.embl.de/), Conserved Domain Database (CDD) (https://www.ncbi.nlm.nih.gov/Structure/bwrpsb/bwrpsb.cgi) [56], MARCOIL, and HEASTER (https://applbio.biologie.uni-frankfurt.de/hsf/heatster/analyse.php#menu) [9], the final results were exactly the same as those obtained using the two approaches employed in the present study. Thus, the method employed for constructing wheat-specific HSF HMM files with HSF HMM files (PF00447) in the present study was highly effective and reasonable, and the combination of multiple approaches avoided the failure to identify *TaHsfs*. The gene family member identification strategy presented in this study will greatly facilitate the identification of HSF family in other species.

A gene family is a group of genes derived from the same ancestor, where two or more copies of the gene may be present. Whole genome duplication is a major driver of gene family expansion to increase the number of gene family members in a single species [57]. Genes with products that have close interactions are usually retained after genome-wide duplication, such as genes involved in signaling pathways, regulatory networks, and the formation of macromolecular complexes. These genes are called dose-sensitive genes, and they include transcription factors and ribonucleoprotein family members [57,58]. If these genes are lost after genome-wide duplication, the metabolic stability of the organism can be disrupted with fatal consequences [59,60]. Segmental and tandem duplications are the other two main causes of the expansion of gene families in plants [61]. Tandem duplication is closely related to the amplification of biotic and abiotic stress-related genes [62,63]. Tandem duplication events only amplify a class of genes, so any redundant dose-sensitive genes produced by these duplication events will disrupt the balance of the related biological pathways. Therefore, these types of duplication events tend to amplify genes at the apical or terminal ends of metabolic pathways as well as dose-insensitive genes [61]. *TaHsfs* are typical dose-sensitive genes because they regulate the expression of HSPs by encoding transcription factors. Thus, during the long process of evolution, wheat has retained 14 groups of complete homeologous triplet HSF gene family members in the ABD sub-genomes, and only a very small number of genes were derived by tandem duplication. This balanced state maintains the metabolic stability of the organism and it is also required for the fine regulation of mechanisms in plants. 

The evolution of gene families is often accompanied by increases or losses of exons, which have played key roles in the evolution of gene families [64]. Thus, we analyzed the number and distribution of exons and introns in each member of the *TaHsf* family and found that the 61 *TaHsfs* contained 1–3 exons and 0–4 introns. The lengths and positions of the *TaHsf* gene introns in the same subclass were relatively well conserved, but the introns varied greatly among the different *TaHsf* subclasses. The presence of introns allows a variety of splicing modes and the same DNA sequence can produce different protein products via alternative splicing of the gene after transcription [65]. This is one explanation for the evolution of the functional diversity of the wheat HSF family and the different responses of *TaHsfs* to temperature [66]. 

In plants, transcription factor HSF*s* are expressed in male reproductive cells during heat stress. In particular, *AtHsfA2* (*AT2G26150*) and *AtHsfA5* (*AT4G13980*) were identified as critical during pollen reproduction in *Arabidopsis* [67,68]. Five class A *Hsfs* and three class B *Hsfs* are up-regulated in rice spikes [40]. In addition, increased levels of *HsfA2* and *HsfA3* were detected during tomato pollen development, and other *Hsfs* were also studied in the anthers [27,41,50]. The molecular roles of *Hsfs* in anther development are not yet known but their downstream regulated HSPs are important molecular chaperones involved in the development of male gametophytes in plants [69,70,71]. Chen et al. showed that cytoplasmic male sterility in sorghum was caused by insufficient mitochondria. When the sterile sorghum line 3A was heated for 4 h, the nuclear-encoded protein HSP70 was expressed in the mitochondria, which promoted an increase in the mitochondria content from 7 mg/g (mitochondria weight/fresh ear weight) to 19 mg/g, and the sterile plants were transformed into fertile plants. However, the mitochondria content was more than 19 mg/g in the maintainer line 3B and it was fertile regardless of whether it was subjected to heat treatment. Thus, HSP70 could regulate fertility by increasing the content of mitochondria to meet the demand for large amounts of energy during pollen development. According to this hypothesis, a rice *Hsp70* antisense expression vector driven by the anther-specific promoter Osg6B was constructed to inhibit *Hsp70* expression during pollen development and block male gamete development, thereby resulting in male sterility [45]. Frank et al. [41] and Giorno et al. [27] demonstrated the high expression levels of *HsfA2* at 42 °C and 36 °C in tomato microspores, respectively, and we demonstrated the high expression level of *TaHsfA2* in fertile anthers cultured at 22 °C. These studies were based on heat treatment, but we hypothesize that increased temperature can induce the upregulation of HSFs to regulate the expression of downstream HSPs in the cytoplasm and increase the number of mitochondria to ensure the energy supply is adequate for pollen development. Thus, the findings obtained in the present study indicate likely roles of wheat HSF family members in anther development, and provide a new theoretical basis for exploring the mechanism responsible for male sterility in wheat. 

## 4. Materials and Methods 

### 4.1. Plant Materials

KTM3315A is a type of thermo-sensitive male sterile wheat with various advantages and it was selected by our research group in 2001. It is completely male sterile during the normal wheat-growing season, whereas its fertility can be restored in a high-temperature environment [37]. Ten pots of KTM3315A were placed in two artificial climate incubators until the flowering stage and they were treated at these conditions: 14 h light (day) and 10 h dark (night) and two different temperatures (day/night temperatures of 17 °C/15 °C for the sterile conditions and 22 °C/20 °C for the fertile conditions). RNA was extracted from the anthers of sterile (AS1, AS2, and AS3) and fertile (AF1, AF2, and AF3) plants during the uninucleate, binucleate, and trinucleate stages, and six samples were used for RNA-seq and qRT-PCR.

### 4.2. Genome-Wide Identification of HSF Family Members in Wheat

The latest wheat reference genome and the HMM profile (PF00447) of the HSF family were downloaded from the wheat genome database IWGSC (IWGSC RefSeq annotation v1.1, https://wheat-urgi.versailles.inra.fr/Seq-Repository/Annotations) [44] and Pfam database (http://pfam.xfam.org/family/PF00447) [72]. The HSF HMM profile was searched in the wheat genome with the hmmsearch program in HMMER software [73] and reliable results were screened based on an E-value less than or equal to 1 × 10^−20^. The results obtained were used to construct a wheat-specific HSF HMM profile by hmmbuild program, before searching the wheat reference genome again with an E-value threshold of 1 × 10^−3^. In order to avoid missing other HSF members, the HSF protein sequences for rice and *Arabidopsis* were downloaded from phytozome (https://phytozome.jgi.doe.gov/pz/portal.html) and used as queries to BLAST the wheat reference genome, where the E-value threshold was 1 × 10^−10^. The results obtained were identified as wheat candidate genes and verified by online software CD-search, SMART, and pfam for presenece of protein domain. Coiled-coil structures were detected using the MARCOIL program. Protein sequences that lacked the DBD domain or coiled-coil structure were removed. All high confidence level HSF protein sequences were submitted to Expasy (https://www.expasy.org/structural_bioinformatics) to calculate the number of amino acids, molecular weight, and theoretical isoelectric point. NetNES (http://www.cbs.dtu.dk/services/NetNES/), cNLS Mapper (http://nls-mapper.iab.keio.ac.jp/cgi-bin/NLS_Mapper_form.cgi#opennewwindow), and HEATSTER were used to search for the NESs and NLSs in the TaHsfs [9,74,75]. The gene annotation information for the *TaHsfs* was submitted to the online site MapGene2 Chrom web V2 (http://mg2c.iask.in/mg2c_v2.0/) to map their chromosomal locations.

### 4.3. Multiple Sequence Alignment, Phylogenetic Analysis, and Classification of TaHsfs

The full-length amino acid sequences of the HSFs in rice and *Arabidopsis* were used for evolutionary analysis with the full-length amino acid sequences of the TaHsfs [13]. All of the sequences were imported into MEGA7 for sequence comparison using the default parameters, and then a phylogenetic tree was constructed using the conserved DBD and OD domains and the connection between them. The phylogenetic tree was constructed using the neighbor-joining method and verified based on the maximum likelihood method, where the parameters were set to the Poisson distribution mode and the bootstrap tests were conducted with 1000 replicates. The classifications of HSFs in wheat were based on the topology of the phylogenetic tree and the classification of HSFs in the other two plants.

### 4.4. Analysis of Gene Duplication and Synteny Relationships of TaHsfs

Segmental and tandem duplications were determined for the *TaHsfs* using McscanX software [76]. Sequences on the same chromosome with a shared similarity greater than 75% and fragment lengths in the alignment that exceeded 75% of the length of the longer sequence were confirmed as tandem duplications. The KA/KS values were calculated for the tandem duplications using KAKS_calculator 2.0 [77]. A segmental duplication map of the genome was prepared using Circos software [78]. In addition to rice and *Arabidopsis*, the genome-wide CDS sequences and genome annotation information for Chinese cabbage (http://brassicadb.org/brad/datasets/pub/Genomes/Brassica_rapa/V1.0/Scaffold1.0/), sorghum (http://www.plantgdb.org/BdGDB/), maize (http://interface.maizegdb.org/gbrowse/maize_v4), and *Brachypodium* (https://phytozome.jgi.doe.gov/pz/portal. html#!info?alias=Org_BdistachyonBd21_3_er) were obtained, and the synteny relationships between the HSF family member in wheat and these species were determined using Mcscanx.

### 4.5. Structural Characterization, Conserved Motifs, and Construction of the Interaction Network for TaHsfs

ClustalW was used to align the conserved DBD and HR-A/B domains in the TaHsfs. The gene structures of the *TaHsfs* were visualized using the online drawing tool Gene Structure Display Server 2.0 (GSDS, http://gsds.cbi.pku.edu.cn/) [79]. The MEME software [80] obtained 15 conserved motifs in the TaHsfs with lengths of 6–50 aa. The gene structure and motifs in the *TaHsfs* were visualized using TB tools [81]. STRING was employed to obtain the regulatory networks between *TaHsfs* and their interacting genes, which were visualized with Cytoscape (https://cytoscape.org/) [82,83,84].

### 4.6. RNA-seq Data Analysis

Part of the wheat transcriptome data was obtained from the Wheat Expression Browser powered by expVIP (http://www.wheat-expression.com/) to study the expression patterns of the *TaHsfs*. The RNA-seq data were derived from nine different growth periods and tissues in Chinese Spring under normal conditions (leaves and shoots in seedling stage, roots in seedling stage, leaves and shoots in vegetative stage, roots in vegetative stage, leaves and shoots in reproductive stage, roots in reproductive stage, spike in reproductive stage, and grain in reproductive stage) [85], as well as under six types of stress and a normal control in the heat and drought tolerant wheat variety TAM107 (heat stress for 1 h, heat stress for 6 h, drought stress for 1 h, drought stress for 6 h, combined drought and heat stress for 1 h, combined drought and heat stress for 6 h, and no stress control) [86]. In addition, in order to study the mechanism responsible for regulating *TaHsfs* in thermo-sensitive male sterile wheat, transcriptome data were analyzed from temperature-sensitive materials under different fertility conditions (AF1, AF2, AF3, AS1, AS2, and AS3) [37]. The heat maps were drawn using the heatmap application in omicshare (http://www.omicshare.com/tools/index.php/Home/help/index/html/combinemore).

### 4.7. qRT-PCR Verification

Total RNA extraction and reverse transcription of cDNA were performed using TRIGene and StarScript II First-strand cDNA Synthesis Mix (Genestar), respectively, according to the manufacturers’ instructions. qRT-PCR was conducted with an Applied Biosystems 7500 Real-Time PCR System and 2×RealStar Green Fast Mixture with ROX II according to the operating instructions and program default settings with three replicates per group. ROX II was added to correct fluorescence signal errors between wells during qRT-PCR reactions. The wheat Actin gene was used as an internal reference, the final relative quantitative results were calculated using the 2^–ΔΔCt^ method [87]. At least three replicates of each cDNA sample were used to perform the qRT-PCR of each gene. Primer 5.0 was used to design the specific primers according to the *TaHsf*s sequences (Table S12).

## Figures and Tables

**Table 1:** Homeologous groups in wheat HSF gene family.

Homoeologous Group (A:B:D)	All Wheat Genes	Classes			Number of Groups	Number of Genes	% of Total TaHSFs
		A	B	C			
1:1:1	35.8%	7	4	3	14	42	68.8
1:1:0/1:0:1	13.2%	2	1	1	4	8	13.1
Orphans/singletons	37.1%	1		1	2	2	3.3
Others	-	1		1	2	9	14.8
Total	-	11	5	6	20	61	100

All wheat genes are distribution among homeologous groups in the whole wheat genome according to IWGSC. A, B, and D represent the three wheat sub-genomes. “Others” denotes circumstances other than those in the table above (see Table S4 for details).

## Supplementary Materials

Supplementary materials can be found at https://www.mdpi.com/1422-0067/21/2/608/s1.

## Abbreviations

HSF	Heat shock transcription factor
*Hsf*	HSF gene
*TaHsf*	Wheat HSF gene
DBD	DNA binding domain
OD	Oligomeric domain
