# Supplementary material for: Genome-Wide Investigation of Heat Shock Transcription Factor Family in Wheat (Triticum aestivum L.) and Possible Roles in Anther Development
Source: Int J Mol Sci. 2020 Jan 17;21(2):608. doi: 10.3390/ijms21020608 (PMC7013567; doi:10.3390/ijms21020608)
Supplement: Supplementary file 1 [file ijms-21-00608-s001.zip › supplementary file/Additional file 9 Table S7.docx]

**Additional file 9: Table S7.** Conserved sequence of motifs for *TaHsfs*.

| **Motif** | **Logo** | **E-value** | **Sites** | **Width** | |
| --- | --- | --- | --- | --- | --- |
| 1. | 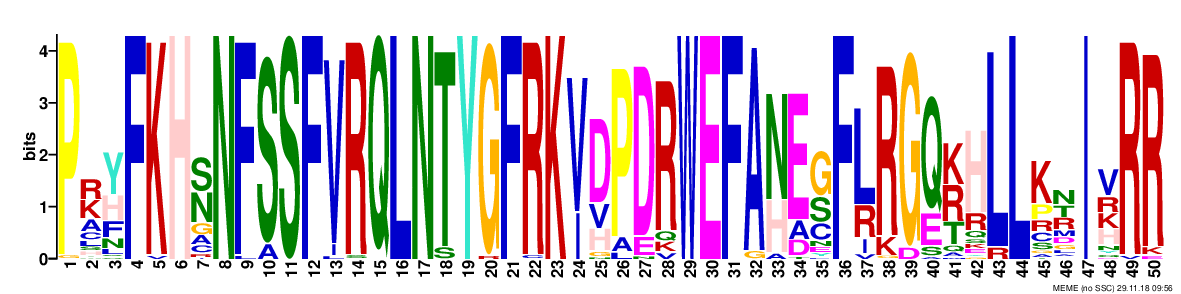 | 7.2e-2131 | 50 | | 50 |
| 2. | 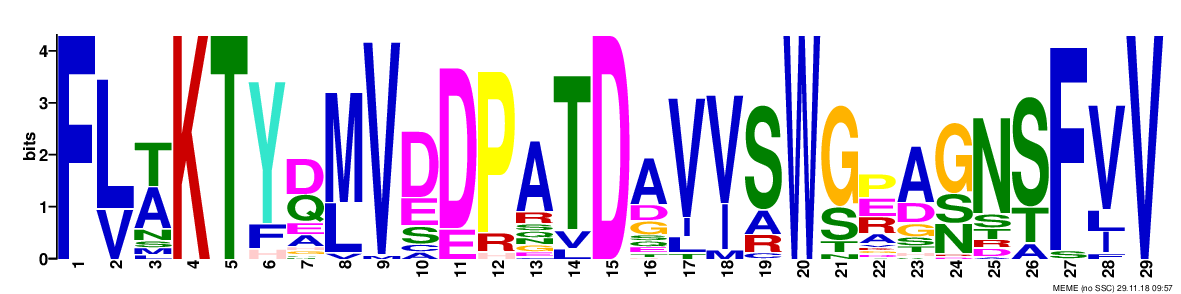 | 1.9e-965 | 50 | | 29 |
| 3. | 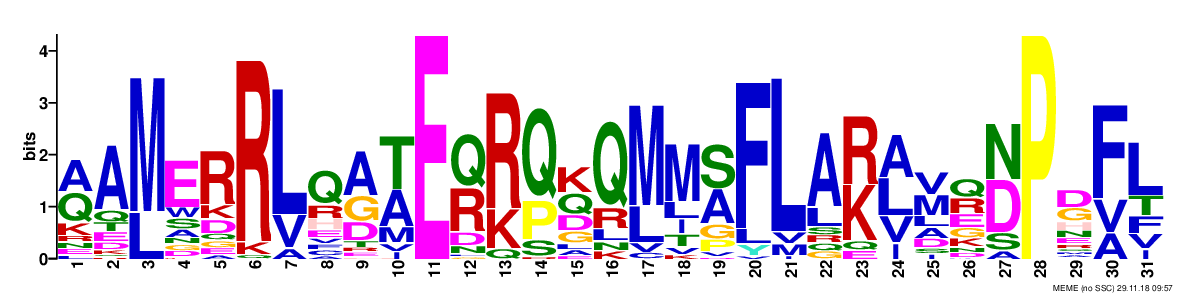 | 3.9e-578 | 46 | | 31 |
| 4. | 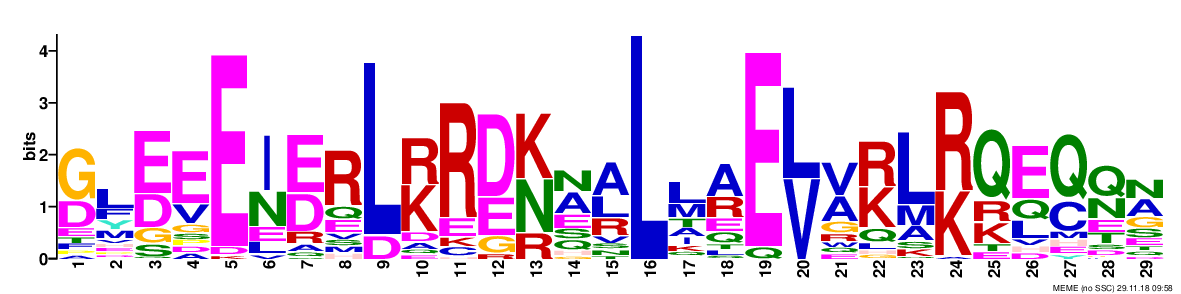 | 4.4e-505 | 49 | | 29 |
| 5. | 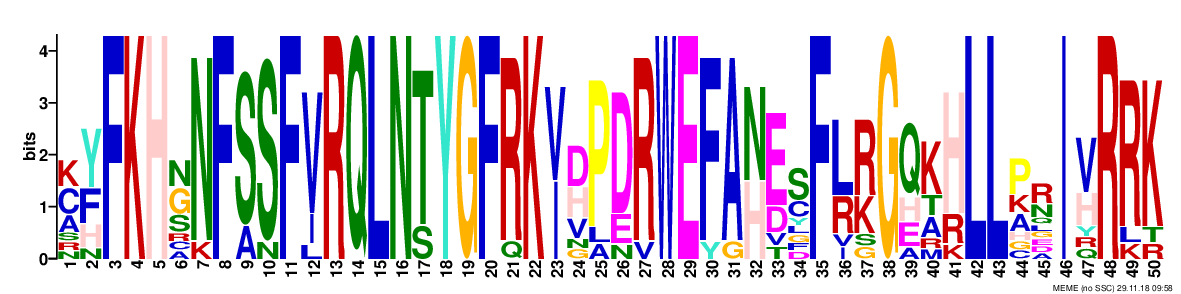 | 4.7e-316 | 11 | | 50 |
| 6. | 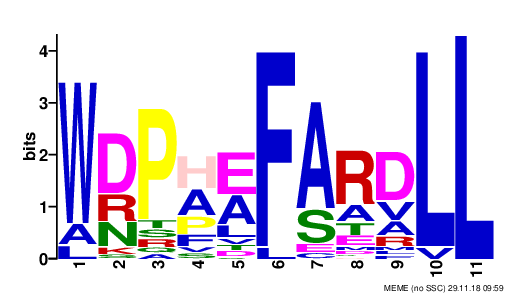 | 5.8e-191 | 50 | | 11 |
| 7. | 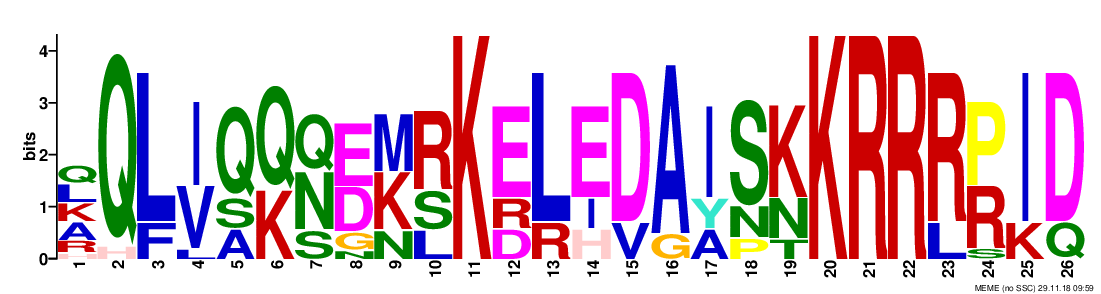 | 2.5e-178 | 15 | | 26 |
| 8. | 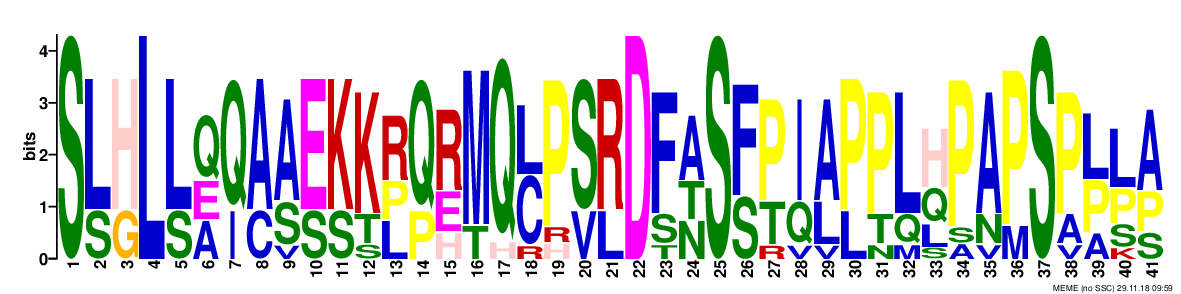 | 3.8e-169 | 11 | | 41 |
| 9. | 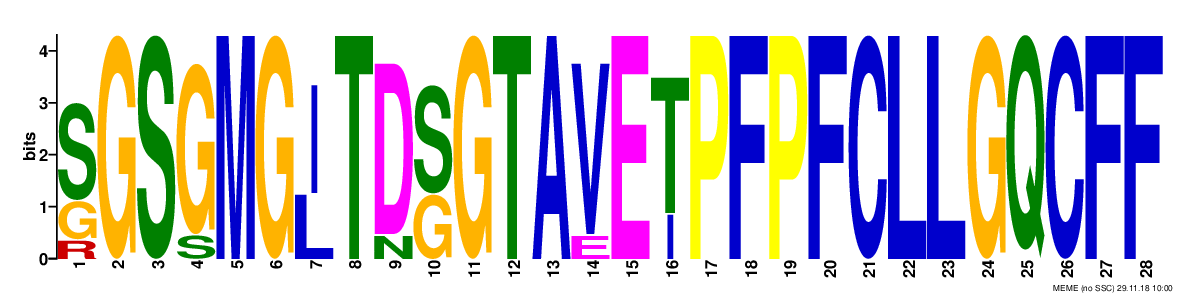 | 8.9e-131 | 8 | | 28 |
| 10. | 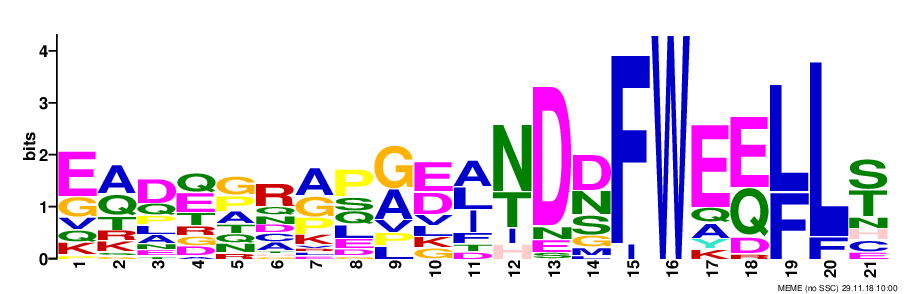 | 2.2e-124 | 26 | | 21 |
| 11. | 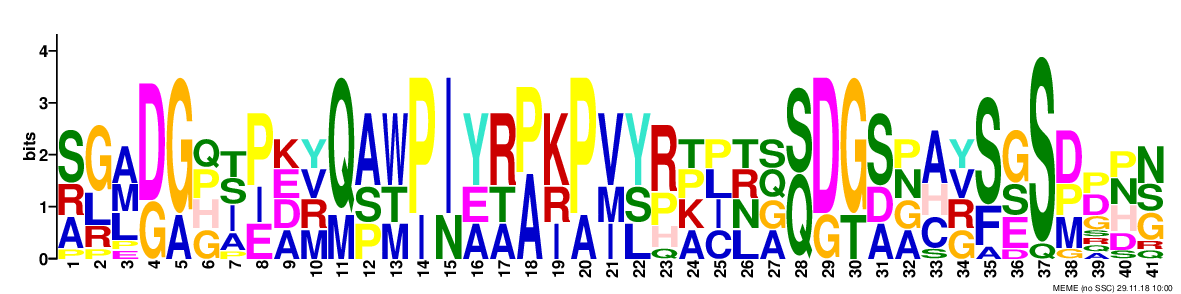 | 1.5e-122 | 12 | | 41 |
| 12. | 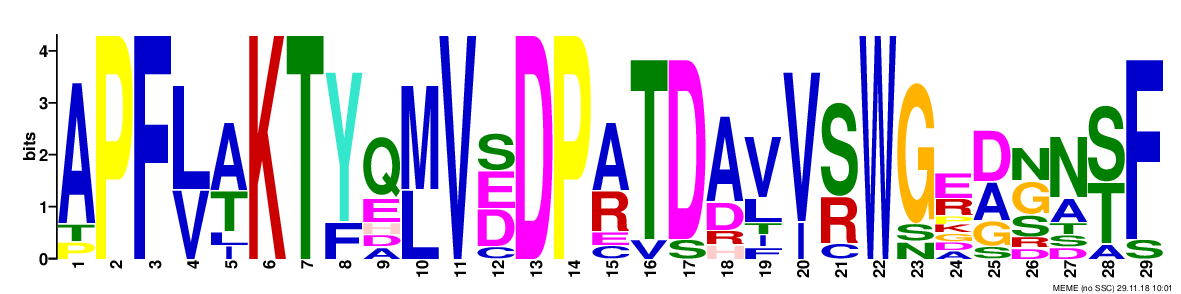 | 1.8e-110 | 10 | | 29 |
| 13. | 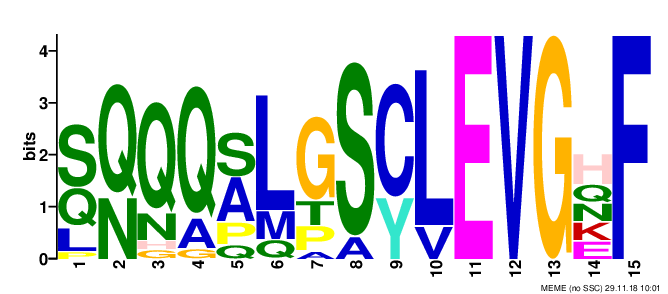 | 4.7e-101 | 17 | | 15 |
| 14. | 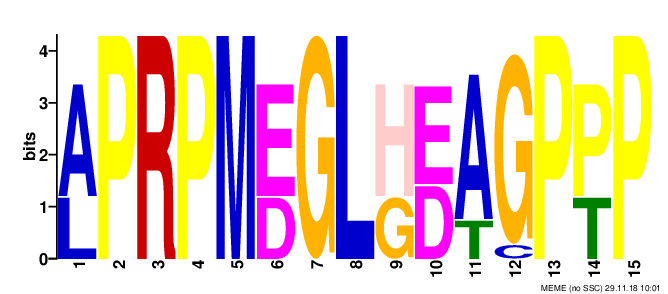 | 3.4e-098 | 14 | | 15 |
| 15. | 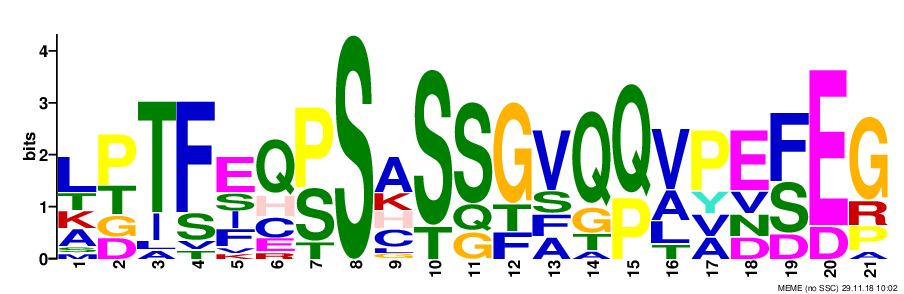 | 5.9e-083 | 17 | | 21 |
