# Supplementary figures and images for: Genome-Wide Investigation of Heat Shock Transcription Factor Family in Wheat (Triticum aestivum L.) and Possible Roles in Anther Development
Source: Int J Mol Sci. 2020 Jan 17;21(2):608. doi: 10.3390/ijms21020608 (PMC7013567; doi:10.3390/ijms21020608)

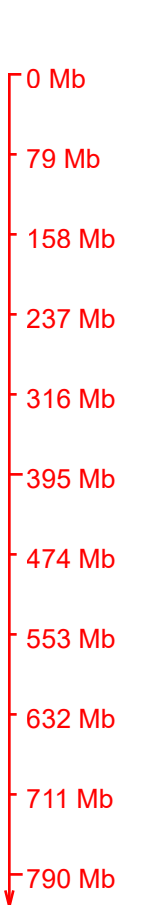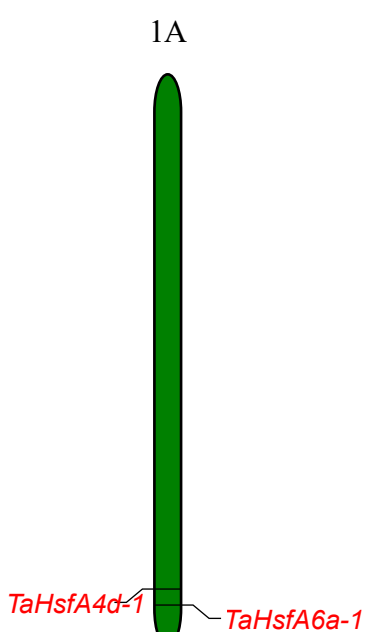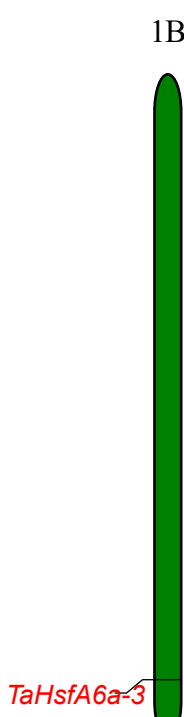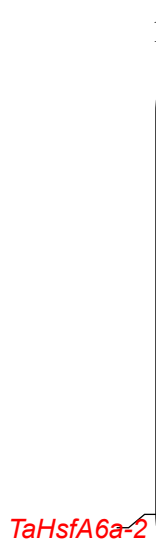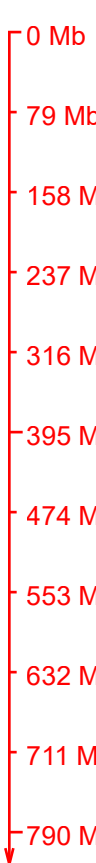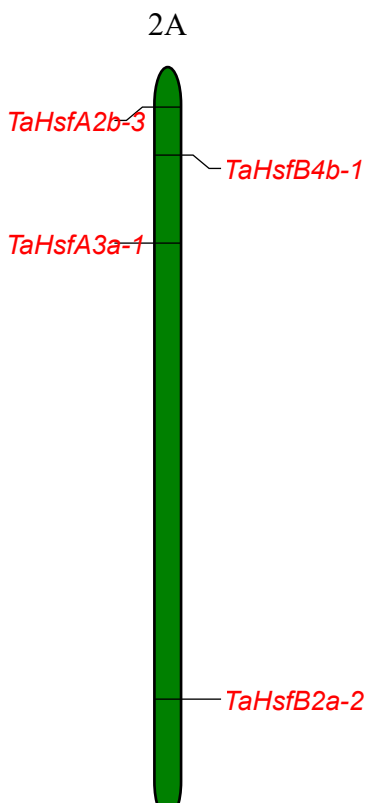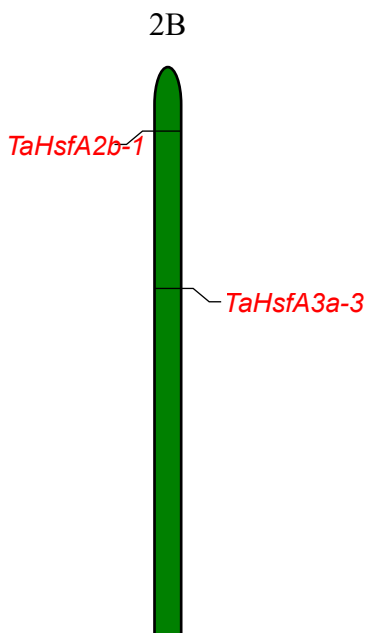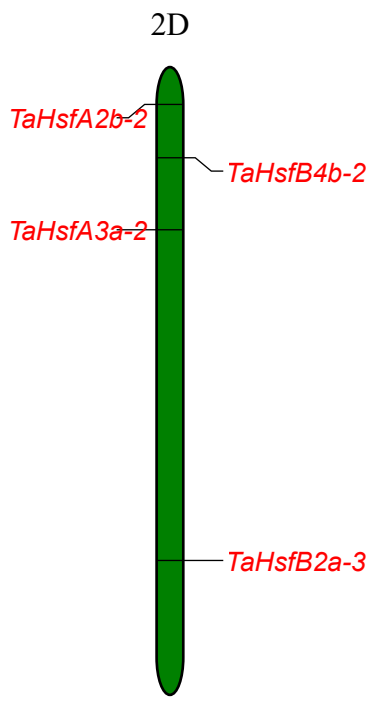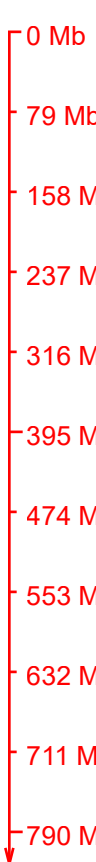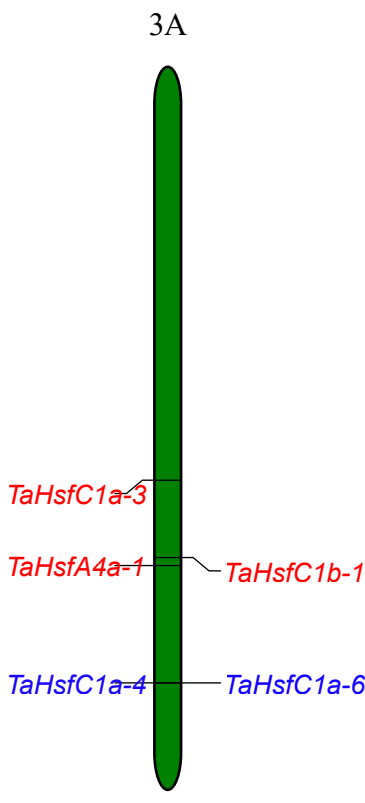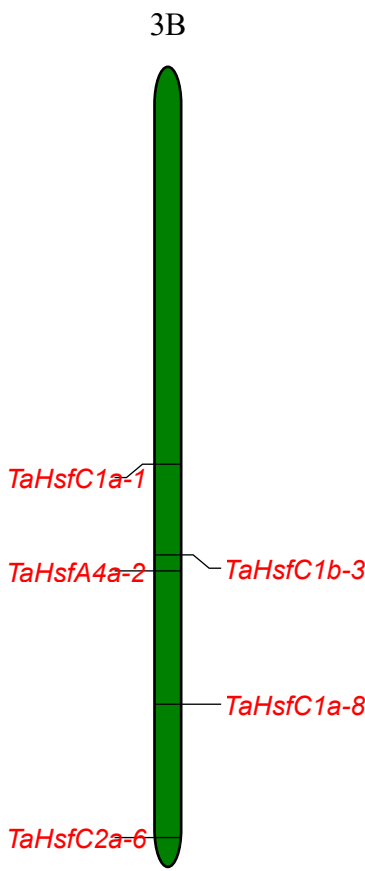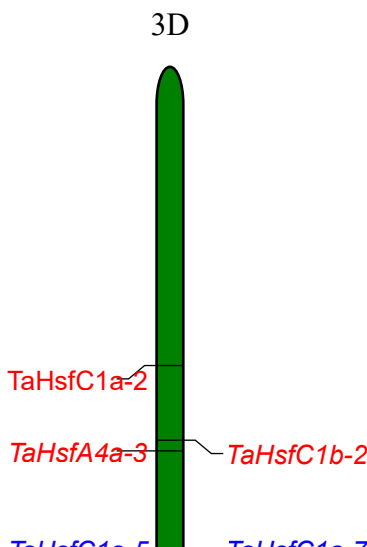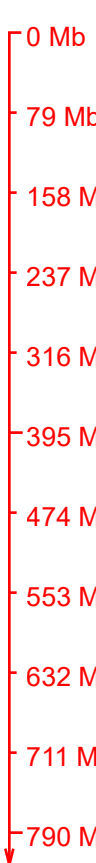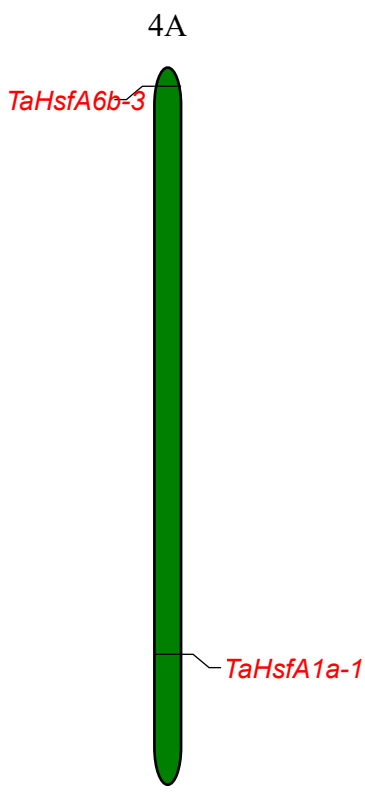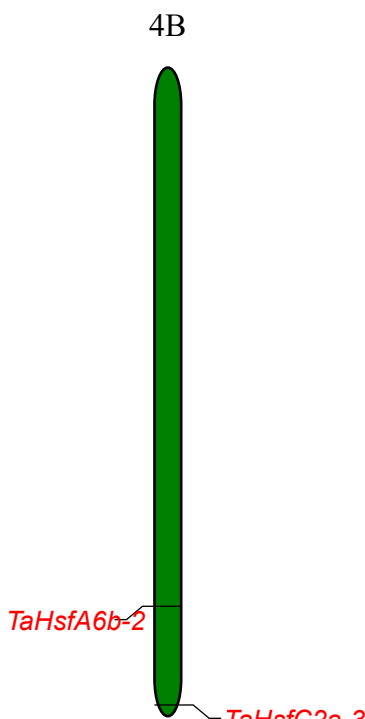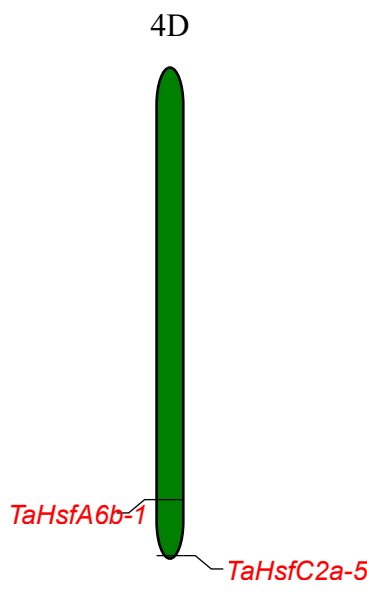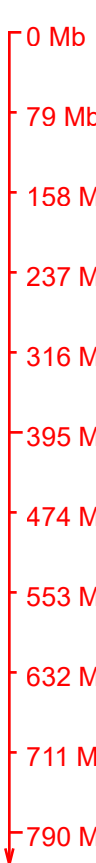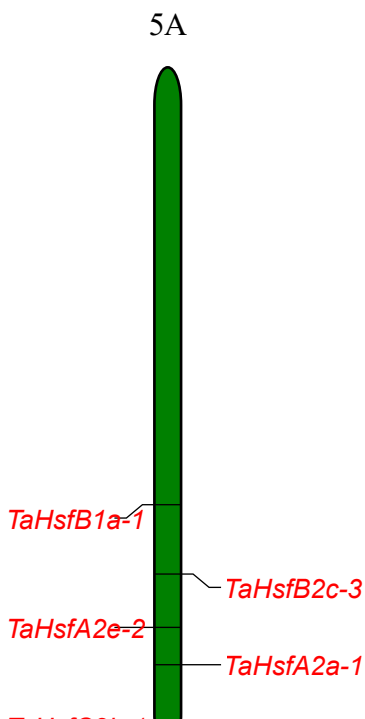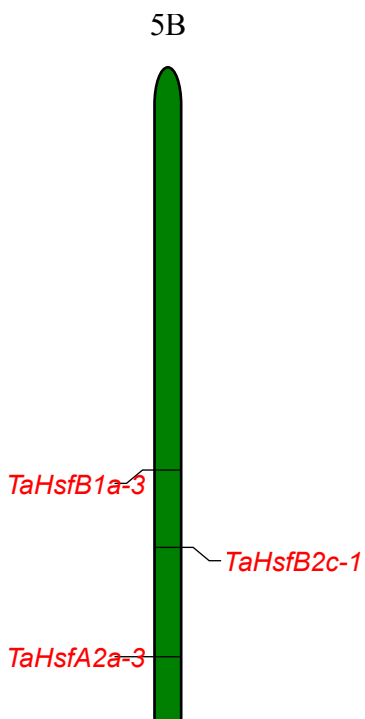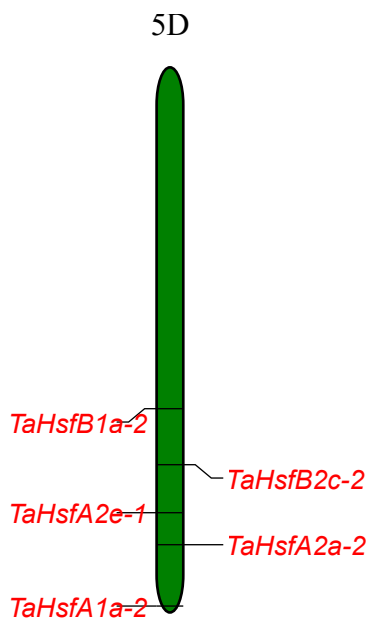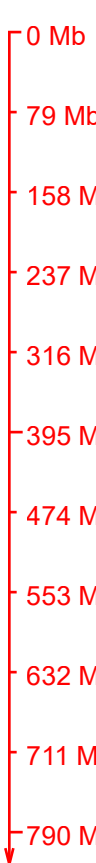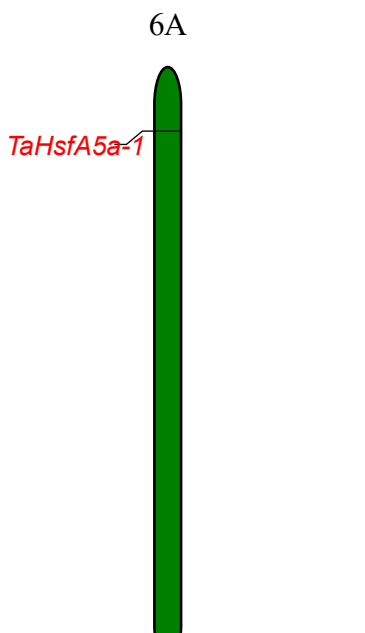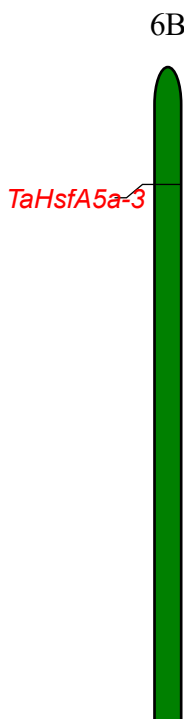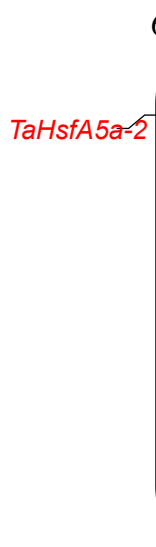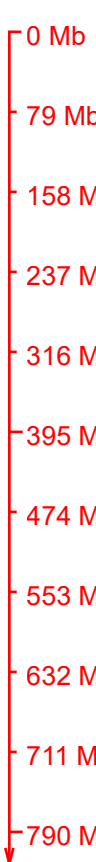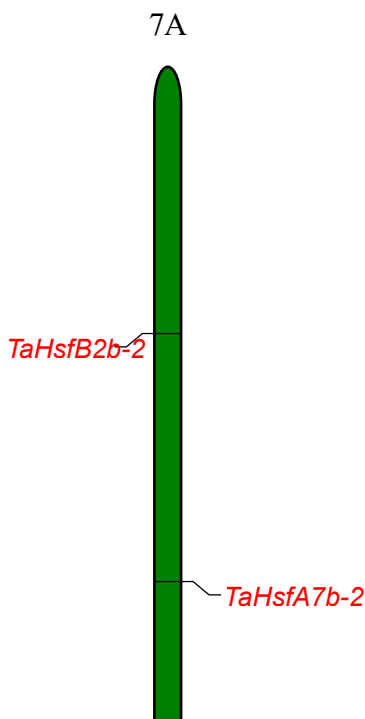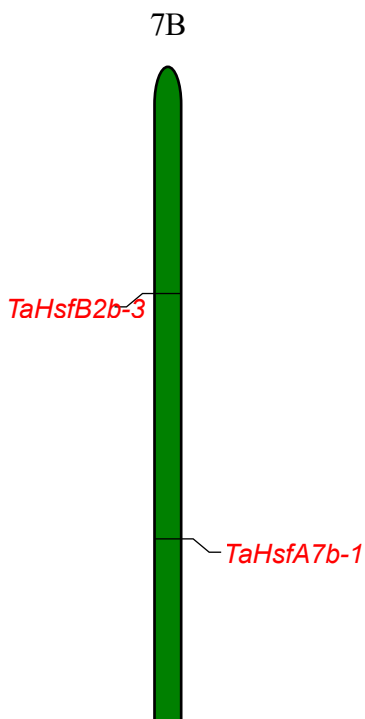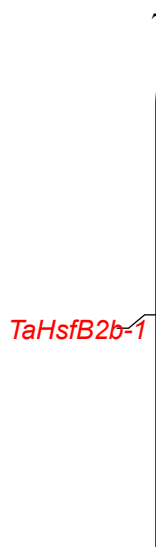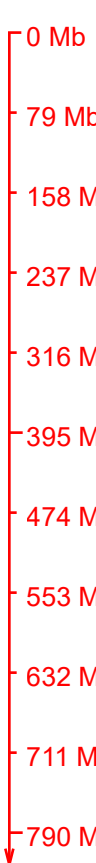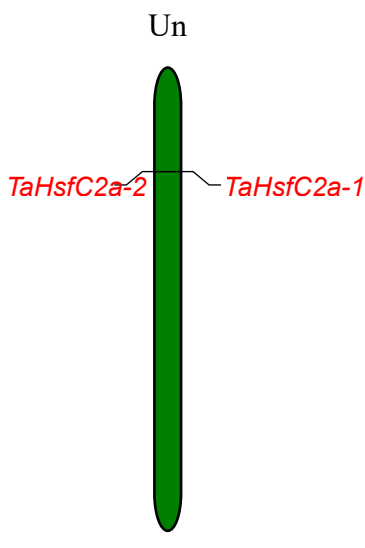

Supplement: Supplementary file 1 [file ijms-21-00608-s001.zip › supplementary file/Additional file 3 Figure S1.pdf]

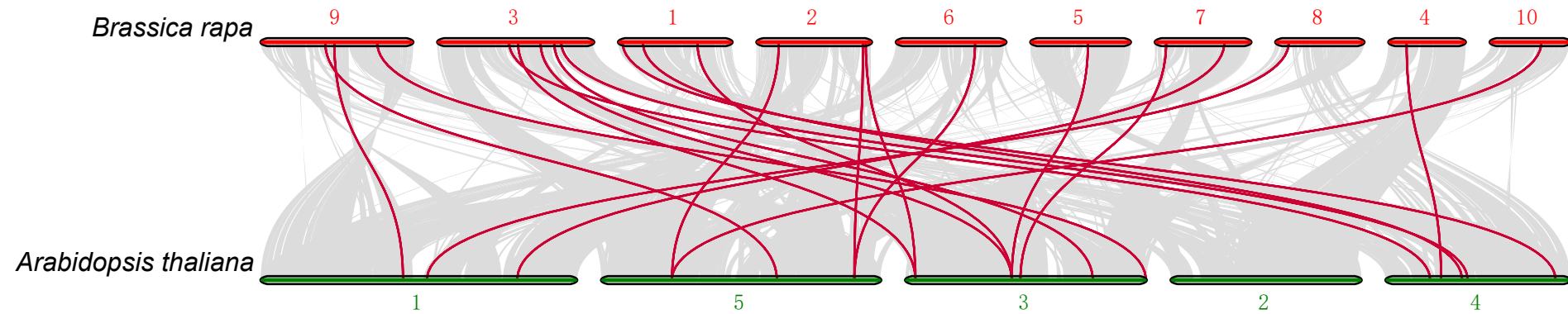

Supplement: Supplementary file 1 [file ijms-21-00608-s001.zip › supplementary file/Additional file 5 Figure S2.pdf]

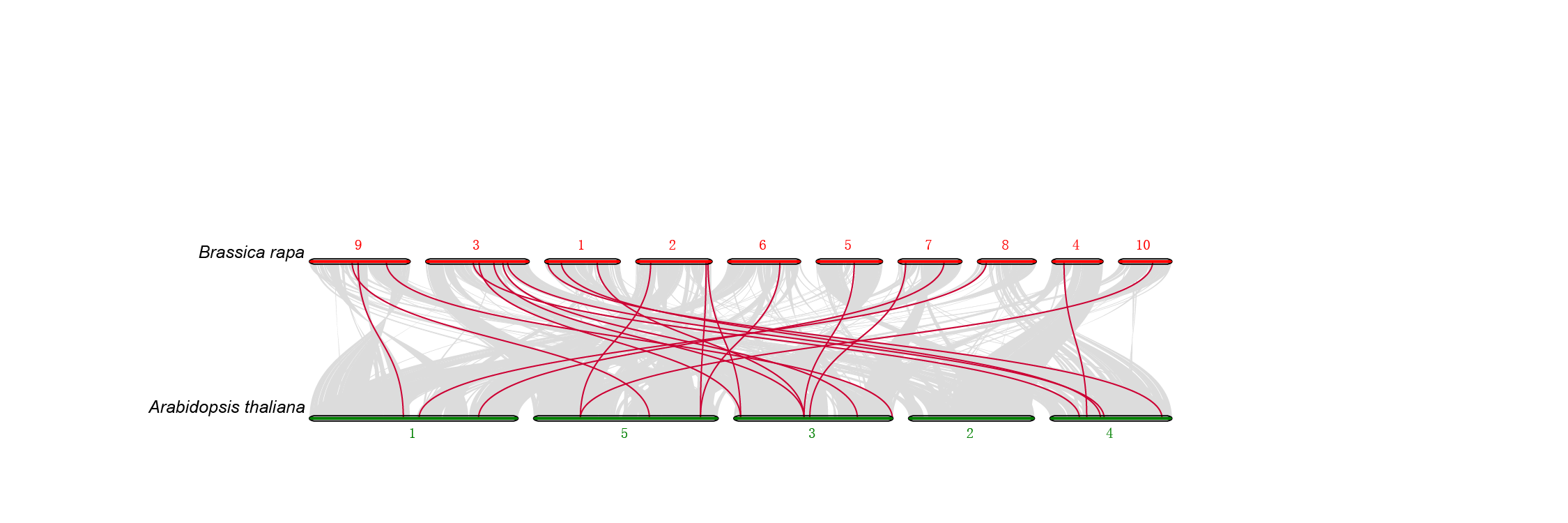

Supplement: Supplementary file 1 [file ijms-21-00608-s001.zip › supplementary file/Additional file 5 Figure S2.png]

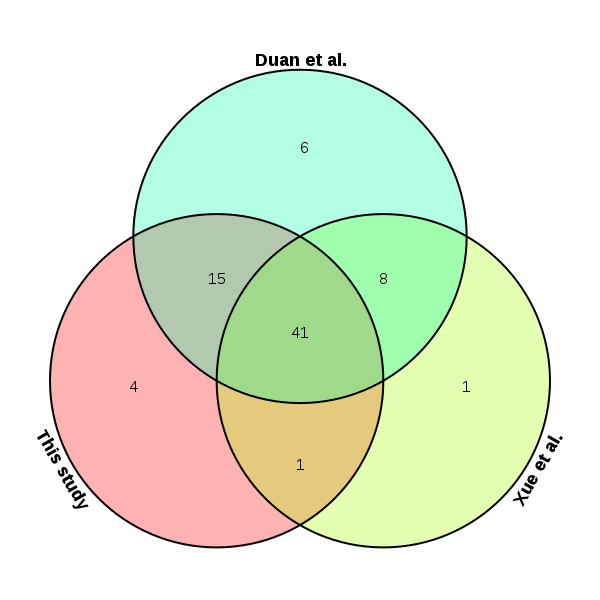

Supplement: Supplementary file 1 [file ijms-21-00608-s001.zip › supplementary file/Additional file13 Figure S3.png]
